# Impact of COVID-19 lockdown on Retinal Surgeries

**DOI:** 10.12669/pjms.37.7.4291

**Published:** 2021

**Authors:** Muhammad Amer Awan, Fiza Shaheen, Fatima Mohsin

**Affiliations:** 1 Dr. Muhammad Amer Awan, Department of Ophthalmology, Shifa International Hospital, Islamabad, Pakistan; 2 Dr. Fiza Shaheen, Department of Ophthalmology, Shifa International Hospital, Islamabad, Pakistan; 3 Dr. Fatima Mohsin. Department of Ophthalmology, Shifa International Hospital, Islamabad, Pakistan

**Keywords:** COVID-19, Eye, Pandemic, Retina, Vitrectomy

## Abstract

**Objectives:**

To determine the frequency and severity of surgical Vitreo-Retinal diseases during COVID-19 lockdown period (LP) and compare it with same period last year.

**Methods:**

Single hospital based retrospective Cohort Study. Data of the patients that underwent retinal surgeries during the COVID-19 LP i.e., 23-03-2020 till 23-06-2020 and same period last year i.e., 23-03-2019 till 23-06-2019 was analyzed.

**Results:**

One hundred thirty-six eyes of 105 patients were included. Among these eyes, 48 (35.3%) were operated during the COVID-19 LP while 88 (64.7%) were operated during the same time last year. A decline of 45.5% (p=0.023) was observed in the frequency of surgeries during the LP. Mean age of patients during the LP was 43.2 ± 20.3 years compared to 48.4 ± 17.9 years last year. There was reduction in the surgeries for Diabetic Tractional Retinal Detachment (11.4% vs 4.2% during LP, p=0.166), Vitreous hemorrhage (10.2 % vs 8.3% during LP, p=0.04), Full thickness macular hole (3.4% vs 0% during LP) and Epiretinal membrane (12.5% vs 0% during LP). While Rhegmatogenous retinal detachment (27.3% vs 58.3% during LP, p<0.001) among other disorders had a higher proportion during the LP.

**Conclusion:**

The decline in the frequency of retinal surgeries during the LP is indicative of complex pathologies presenting later with more advanced disease. However, earlier presentation and an increase trend in surgeries for RRD during the LP shows the positive impact of free time on the health concerns of our population.

## INTRODUCTION

The spread of COVID-19 virus has altered the course of life round the globe. As a safety response to this ongoing pandemic, hospitals all over the world reduced the patient encounter and in person checkups in the outpatient departments in order to maximize the use of resources to the frontlines.^1^,^2^ Ophthalmology has been one of the most severely hit specialty in terms of patient number and surgeries.^3^,^4^ Many specialties utilized Tele communication for keeping an update of their patients. However, for ophthalmology where diagnosis and management are difficult without an elaborate examination of the ocular structures, telemedicine apps were not very helpful.

Where most of the ophthalmic pathologies can be managed electively, there are still quiet notable problems that need to be addressed urgently due to their relatively irreversible adverse effects on vision. These include acute glaucoma, corneal ulcer, ocular trauma and retinal detachment.^5^–^8^ Among the various sub specialties, Vitreo-Retina has to be the most affected area in this aspect because of its dealing with potentially blinding pathologies.^9^

Studies are being conducted throughout the globe to assess the impact of COVID-19 LP on the medical as well as surgical aspect of retina. In literature, a study has recently explored the effects on LP on Retina clinics and intra-vitreal injections^9^ while assessment of acute ophthalmic emergencies^10^ especially rhegmatogenous retinal detachment.^11^,^12^ has also been done just recently.

The objective of our study was to estimate the impact of COVID-19 pandemic specifically in the field of surgical retina where delayed presentations and treatment is considered to affect the visual prognosis. We tend to included all the posterior segment pathologies that underwent surgical intervention instead of focusing on a single pathology in our area. It gave us a broader view of the effects of COVID-19 LP on surgical retina and will be of benefit in sorting measures to handle such situations in a better manner in the near future.

## METHODS

It is a retrospective, cohort study conducted at Shifa International Hospital, Islamabad, Pakistan after taking approval from the institutional review board (IRB) (Ref No: 304-1124-2020, Date of approval; 18-08-2020) of the hospital. Patients were identified using their assigned medical record numbers from the surgical data of the operating room. Those who underwent retinal surgeries during the following time period were included in the study and their records analyzed.

During COVID-19 lockdown period (LP): 23rd March 2020 to 23 June 2020 and it was compared with same time last year i.e., 23rd March 2019 to 23rd June 2019. Collected data included; medical record number, age, gender, laterality, duration of symptoms, diagnosis, procedure, date of procedure and per-operative tamponade.

Data was analyzed using Statistical Package for the Social Sciences (SPSS) 21 version. For qualitative measures, frequency and percentage was used while mean and standard deviation was calculated for quantitative measures. Chi-square test was used to assess the comparison of presentation between the two time periods. One sample *t*-test was used to calculate the level of significance of each pathology. A p-value of <0.05 was considered to be statistically significant.

## RESULTS

One hundred thirty six eyes of 105 patients were included in our data. Among these eyes, 48 (35.3%) were operated during the COVID-19 LP while 88 (64.7%) were operated during the same time last year. A significant decline of 45.5% (n=88 vs 48 during LP, p=0.023) was observed in the frequency of retinal surgeries during the LP.

Mean age of patients during the LP was 43.2± 20.3 years compared to 48.4± 17.9 years last year. There was 12.8% decline in the female patients who had retinal surgeries during the LP [42% (n=37) vs 29.2% (n=14) during LP, p<0.001].

Mean duration of presentation after the onset of symptoms was unexpectedly earlier i.e., 28.51 ± 23.59 days during the LP compared to 69.38 ± 69.9 days last year. There was a decline of 7% in the surgery of scheduled cases of removal of silicon oil during the LP [21.6% (n=19) vs 14.6% (n=7) during LP, p=0.006].

**Fig.1:**
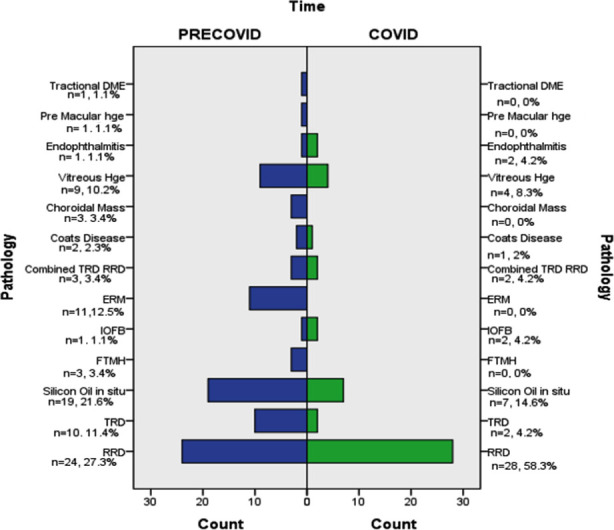
Bar Chart shows comparison of frequency of various pathologies during Pre COVID-19 lockdown and during COVID-19 Lockdown period. (DME=Diabetic macular edema, He=Hemorrhage, RRD=Rhegmatoegnous retinal detachment, TRD=Tractional Retinal Detachment, ERM=Epiretinal membrane, IOFB=Intraocular foreign body, FTMH=Full thickness macular hole).

There was reduction in the surgeries for Diabetic Tractional Retinal Detachment [TRD] [11.4% (n=10) vs 4.2% (n=2) during LP, p=0.166], Vitreous hemorrhage [VH] [10.2 % (n=9) vs 8.3% (n=4) during LP, p=0.04], Full thickness macular hole [FTMH] [3.4% (n=3) vs 0%(n=0) during LP], Epiretinal membrane [ERM] [12.5% (n=11) vs 0% (n=0) during LP] and some other pathologies. While Rhegmatogenous retinal detachment [RRD] [27.3% (n=24) vs 58.3% (n=28) during LP, p<0.001], intra ocular foreign body [IOFB] [1.1% (n=1) vs 4.2% (n=2) during LP, p=0.184] and endophthalmitis [1.1% (n=1) vs 4.2% (n=2) during LP, p=0.184] were among the pathologies with higher proportion during the LP. Increase in frequency of RRD surgeries was statistically significant (p<0.001). A comparison between the frequencies of various pathologies is given in Fig.1.

Among the eyes operated for RRD, there was 10.1% increase in macula off RRD during the LP [79.2% (n=19) vs 89.3% (n=25) during LP] but no significant difference in total RRD between the two periods [33.3% (n=8) vs 28.6% (n=8) during LP] (Table-I). However fewer RRD with proliferative vitreo-retinopathy presented during the LP [45.8% (n=11) vs 25% (n=7) during LP].

**Table I:** Characteristics of Rhegmatogenous Retinal Detachment presenting during the two periods. (PVR=Proliferative vitreoretinopathy, PVR C= Proliferative vitreoretinopathy grade C).

*Characteristics of Retinal Detachment*		*Time*	
		*PRECOVID n= 24*	*COVID n= 28*
Macular Status	Off	19 (79.2%)	25(89.3%)
	On	4(16.7%)	2(7.1%)
	Bisected	1(4.1%)	1(3.6%)
Clock hours of RD	1-3	5(20.8%)	3(10.7%)
	1-6	7(29.1%)	10(35.7%)
	1-9	4(16.7%)	7(25%)
	1-12	8(33.3%)	8(28.6%)
PVR C	None	13(54.2%)	21(75%)
	CP3	5(20.8%)	5(17.8%)
	CP6	4(16.7%)	1(3.6%)
	CP9	1(4.1%)	0
	CP12	1(4.1%)	1(3.6%)

**Fig.2:**
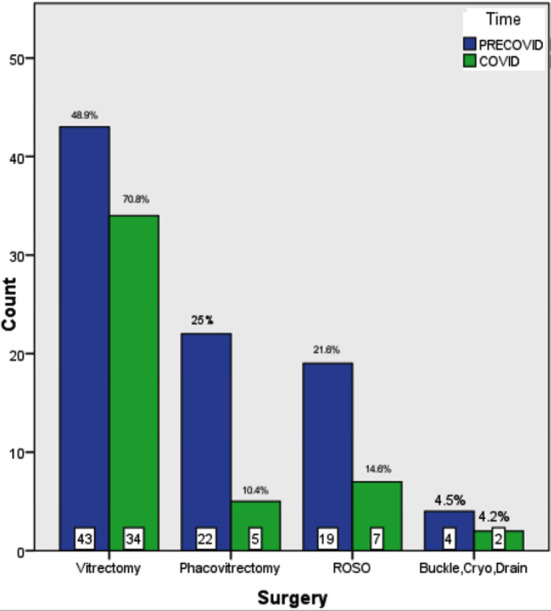
A comparison of various surgeries performed during the pre-lockdown period and during the lockdown period. (ROSO= Removal of Silicon Oil, Buckle Cryo Drain= Scleral Buckling procedure with drainage and cryotherapy)

There was a decreased trend in the combined phacoemulsification cataract and vitrectomy surgery [25% (n=22) vs 10.4% (n=5) during LP, p=0.022]. However, an increase in vitrectomy surgery [48.9 (n=43) vs 70.8% (n=34) during LP, p<0.001] and a decline in the scheduled procedure of removal of silicon oil [21.6% (n=19) vs 14.6% (n=7) during LP, p=0.006] were observed during LP. Scleral buckling procedures were almost same in numbers [4.5% (n=4) vs 4.2% (n=2) during LP, p=0.175]. (Fig.2)

Among the various tamponade agents used during vitrectomy surgery; silicon oil was more frequently utilized during the LP [36.9% (n=31) vs 47.8% (n=22) during LP, p<0.001]. Followed by Hexa-fluoro-ethane [C2F6] gas [6.0% (n=5) vs 17.4% (n=8) during LP, p=0.001] and octa-fluoro-propane [C3F8] gas [1.2% (n=1) vs 8.7% (n=4) during LP, p=0.016] that were more commonly used during the LP while sulfur hexafluoride [SF6] gas [8.3% (n=7) vs 6.5%(n=3) during LP, p=0.081] was less frequently utilized in comparison to the last year. Densiron oil was not used in any eye during LP [2.4% (n=2) vs 0% (n=0) during LP].

## DISCUSSION

In our study, we report a significant decline in the frequency of retinal surgeries during the COVID-19 LP in Pakistan. A 45.5% reduction of surgeries compared to the same period last year reflects a significant burden as well as presentation of more severe disease in the later months.

More presentation of younger patients during the LP suggests that people had more time to pay attention to their health issues than previously owing to the closing of schools and offices. This observation is further supported by an earlier presentation from the onset of symptoms during the LP. However, a 13% decline in the female presentation compared to same time last year is rather an alarming situation. In our society where female issues are not addressed as seriously as that of male, COVID-19 LP might have further worsened the situation.

Being a third world country due to lack of facilities and screening programs, diabetic retinopathy usually presents as an advanced disease requiring surgical intervention. A significant decline in the surgery of VH [10.2 % (n=9) vs 8.3% (n=4) during LP, p=0.04] and diabetic TRD including combined TRD and RRD [14.7 % (TRD n=10, Combined TRD and RRD=3) vs 8.3% (TRD n=2, Combined TRD and RRD=2) during LP, p=0.04] during the LP is a matter of great concern and is predictive of a greater incidence of visual loss due to advanced diabetic eye disease this year in our country.

While most retinal pathologies suffered a reduction of surgeries during the LP, we have observed a 14.2% rise in the RRD surgeries consistent with the rise reported by Akram et al. in his survey involving 85 vitreo-retinal units in UK.^11^ This might be partly due to our facility being the only tertiary care unit offering the services of urgent retinal surgeries in our region during this unprecedented time. This increase is in contrast to a 62% decline reported in UK in a recent analysis as well as two other studies conducted recently.^10^,^12^,^13^ Among the key features of RRD; more macular off status was observed during LP in our study that is consistent with previous studies.^13^,^14^ However, we report almost same frequency of total RRD surgeries in LP and fewer RRD with PVR in our region in contrast to recently reported results in UK^13^,^14^ as well as USA^15^ and Canada.^16^ These results are also in favor of our observation of people taking their acute health concerns more seriously during the LP than before. A comparison of various features of RRD with recently reported studies are given in Table-II. There was an increase in the utilization of silicon oil tamponade and long-acting gas (C3F8) in various vitreo-retinal pathologies during the LP. This trend is similar to the results noted previously as well.^14^ We noted no significant difference in scleral buckling procedure during LP and last year.

**Table II:** Comparison of various features of RRD reported in other recently published studies; NA = not available, n = number, RRD = Rhegmatogenous retinal detachment, PVR = proliferative vitreo-retinopathy, LP = lockdown period.

*Study*	*Frequency of RRD*	*Age (Mean years)*	*Time of presentation (Mean days)*	*On Status of Macula (Percentage frequency)*	*PVR*	*Result*
Shams et al.^12^	n=43 vs 86 (weekly) before LP	NA	NA	34.9 vs 44.2% before LP	NA	Decline in presentation
Jasani et al.^13^	n=46 vs 86 before LP	NA	NA	32.6 vs 47.7% before LP	8.7 vs 2.3% before LP	Decline in presentation
Awad et al.^14^	n= 37 vs 92 before LP	NA	14.8 vs 22.5 before LP	32.4 vs 47.8 % before LP	24.3 vs 9.8% before LP	Decline in presentation
Patel et al.^15^	n=82 vs 111 before LP	58.5 vs 59.0 before LP	5.5 vs 4 before LP	24.4 vs 49.5% before LP	13.4 vs 4.5% before LP	Decline in presentation
Arjamand et al.^16^	n= 87 vs 100 before LP	57.79 vs 56.02 before LP	18.89 vs 17.26 before LP	29.9 vs 49 % before LP	NA	Decline in presentation
This study	n= 28 vs 24 before LP	43.2 vs 48.4 before LP	28.51 vs 69.38 before LP	7.1 vs 16.7% before LP	25 vs 45.8% before LP	Increase in presentation

Telemedicine is being progressively utilized by various medical specialties all over the world.^17^-^20^ with various programs for ophthalmology^21^,^22^ including a model for the surveillance of diabetic eye diseases as well.^23^ A survey conducted in India recently also shows that for majority of day to day ophthalmological practice, telephonic communication was utilized by most of the ophthalmologists.^24^ However, its role in the diagnosis and management of vitreo-retinal pathologies that require an elaborate retinal examination with utilization of various imaging tools is quiet questionable.

### Limitations of the study

To date, our study is a first of its kind that has reported the impact of COVID-19 on the surgical retina from our region. It is limited by its retrospective nature and in assessment of the effect of LP focused on only one aspect and sub specialty of ophthalmology. While a larger study assessing the frequency of outpatient department visits, other minor ophthalmic procedures and anterior segment surgeries is also required in order to evaluate the collective impact of COVID-19 on the overall ophthalmological practice.

## CONCLUSION

Ophthalmology being a specialty where direct patient interaction and examination is pivotal for the diagnosis and management is one of the hardest hit specialties during COVID-19 pandemic. The decline in the frequency of retinal surgeries during the LP is indicative of complex pathologies presenting later with more advanced disease and increasing the work load. A significant reduction in advanced diabetic eye disease surgeries during the LP may lead to poor visual outcomes in these patients during the upcoming times. However, earlier presentation and an increase trend in surgeries for RRD during the LP shows the positive impact of free time on the health concerns of our population.

### Authors Contribution:

**MAA:** Conceived, designed and did statistical analysis & editing of manuscript. He is responsible for the integrity and authenticity of work.

**FS:** Did data collection and manuscript writing.

**FM:** Did review and final approval of manuscript.
